# Gp78, an E3 Ubiquitin Ligase Acts as a Gatekeeper Suppressing Nonalcoholic Steatohepatitis (NASH) and Liver Cancer

**DOI:** 10.1371/journal.pone.0118448

**Published:** 2015-03-19

**Authors:** Tianpeng Zhang, Dhong Hyo Kho, Ying Wang, Yosuke Harazono, Kosei Nakajima, Youming Xie, Avraham Raz

**Affiliations:** Departments of Oncology and Pathology, Wayne State University School of Medicine and the Karmanos Cancer Institute, Detroit, Michigan, United States of America; H. Lee Moffitt Cancer Center & Research Institute, UNITED STATES

## Abstract

Nonalcoholic steatohepatitis (NASH) is related to metabolic dysregulation and the perturbation of endoplasmic reticulum (ER) homeostasis that frequently develops into hepatocellular carcinoma (HCC). Gp78 is E3 ligase, which regulates endoplasmic reticulum-associated degradation (ERAD) by ubiquitinylation of misfolded ER proteins. Here, we report that upon ageing (12 months), gp78^-/-^ mice developed obesity, recapitulating age-related human NASH. Liver histology of gp78^-/-^ mice revealed typical steatosis, hepatic inflammation and fibrosis, followed by progression to hepatocellular tumors. Acute ER stress revealed that loss of gp78 results in up regulation of unfolded protein response (UPR) pathways and SREBP-1 regulating *de novo* lipogenesis, responsible for fatty liver. Tissue array of human hepatocellular carcinoma (HCC) demonstrated that the expression of gp78 was inversely correlated with clinical grades of cancer. Here, we have described the generation of the first preclinical experimental model system which spontaneously develops age-related NASH and HCC, linking ERAD to hepatosteatosis, cirrhosis, and cancer. It suggests that gp78 is a regulator of normal liver homeostasis and a tumor suppressor in human liver.

## Introduction

Nonalcoholic fatty liver disease (NAFLD) is the most predominant hepatic manifestation of the metabolic syndrome and is a disease with multiple characteristics including simple steatosis and nonalcoholic steatohepatitis (NASH). NASH is characterized by excess fat in the liver, inflammation, injury and fibrosis, which can progress to cirrhosis and hepatocellular carcinoma (HCC) [1,2]. Steatosis is defined as the presence of hepatic triglyceride (TG) droplets in more than 5% of hepatocytes [2]. This scenario from NASH to cancer has not been conclusively determined, although previous mouse models were created to recapitulate features of a human disease continuum [2,3]. In molecular mechanisms underlying NAFLD, the endoplasmic reticulum (ER) stress response has recently been proposed to play a crucial role [4,5]. The endoplasmic reticulum (ER) is a membranous network responsible for synthesis, maturation, and protein sorting to the plasma membrane or extracellular *milieu* [6]. The unfolded protein response (UPR) is activated to cope with pathophysiological agents or conditions to elicit ER stress by reducing protein synthesis, facilitating protein degradation, and increasing production of chaperones and foldases that guide nascent or misfolded protein to fold correctly [7]. Three major arms of UPR are evolutionarily conserved from yeast to metazoans and act as proximal sensors of ER stress, which are membrane-spanning proteins including activating transcription factor 6 (ATF6), inositol-requiring enzyme 1 (IRE1), and double-stranded RNA-activated protein kinase-like ER kinase (PERK) [7]. If misfolded proteins are not functionally resolved, they are translocated from the endoplasmic reticulum (ER) to the cytosol where they are degraded by the ubiquitin-proteasome machinery, known as ER-associated degradation (ERAD) [8,9]. However, delayed or insufficient UPR may turn physiological homeostasis to pathophysiological outcomes, including fat accumulation, inflammation, fibrosis and apoptosis, systemically leading to chronic metabolic diseases such as obesity, insulin resistance, and type 2 diabetes [6]. Linkage of UPR pathways to the prevention of steatosis has been elucidated in knockout mice which are disrupted by a single UPR sensor arm or immediate downstream gene, leading to hepatic steatosis such as knockout mice of ATF6α, liver specific Ire1αand GRP78 chaperone [10]. Mechanisms underlying ER stress-induced steatosis include activation of the sterol regulatory element-binding proteins (SREBPs); transcription factors involved in *de novo* lipid biosynthesis. Elevated SREBP-1c correlates with hepatic steatosis in human NAFLD patients [11]. During prolonged stress, the role of C/EBP homologous protein (CHOP) in ER stress-induced apoptosis was illustrated in Chop^−/−^ mice in which CHOP deficiency provides partial resistance to ER stress-induced apoptosis [12]. The contribution of UPR deficiency and prolonged ER stress to the pathogenesis of HCC has been elucidated in chemical carcinogen-induced CHOP knockout mice and induction of CHOP is frequently observed in transposon-induced liver tumors [13].

ERAD is often viewed as a constitutive process due to the sporadic errors, which may occur during the synthesis and folding of proteins. It also regulates the turnover of certain folded proteins regulating metabolism [14]. It couples with UPR by eliminating misfolded proteins and for this reason, genes of ERAD machinery are up-regulated by ER stress and UPR pathways [15]. Genetic ablation of a number of ERAD components leads to embryonic lethality in mice of Hrd1/synoviolin E3 ligase and p97 (VCP/CDC48) ATPase [16,17], which highlights the functional necessity of ERAD. Thus, deficiency of ERAD are proposed to culminate in the accumulation of misfolded proteins in the lumen and membrane of the ER, a condition known as chronic ER stress, which is common to several diseases [18].

gp78 was at first identified as a receptor of autocrine motility factor (AMFR) and was subsequently characterized as a RING-dependent and ER membrane-anchored E3 ligase whose catalytic domains reside in the cytoplasm [19]. It also possesses multiple membrane spanning domains, involved in ubiquitination-mediated degradation of various substrates, including CD3-δ, ApoB lipoprotein, cystic fibrosis transmembrane conductance regulator (CFTR), and the metastasis suppressor KAI1 [20,21]. Others have reported that gp78 might ubiquitinate certain folded proteins and may function as a metabolic regulator of genes such as HMG-CoA reductase (HMGCR) and Insigs involved in lipid metabolism. On the other hand, given the critical role of gp78 in ERAD-regulating ER homeostasis, it is conceivable that a defect of gp78-mediated ERAD processes can lead to chronic ER stress and has a significant impact on cell viability, particularly for cells bearing a heavy burden of misfolded proteins and might be associated with NASH.

The purpose of the study was to examine the spontaneous phenotype of gp78-knockout mice exposed to normal dietary conditions. We have found an age-related gp78 role associated with dysregulation of metabolism and ER homeostasis in the liver of gp78 knockout mice. Genetic disruption of gp78 in mice developed fatty liver, inflammation and spontaneous hepatocellular cancer in aged mice.

## Results

### Construction of gp78-KO mouse and gp78 up regulation in response to ER stress

**Fig 1:**
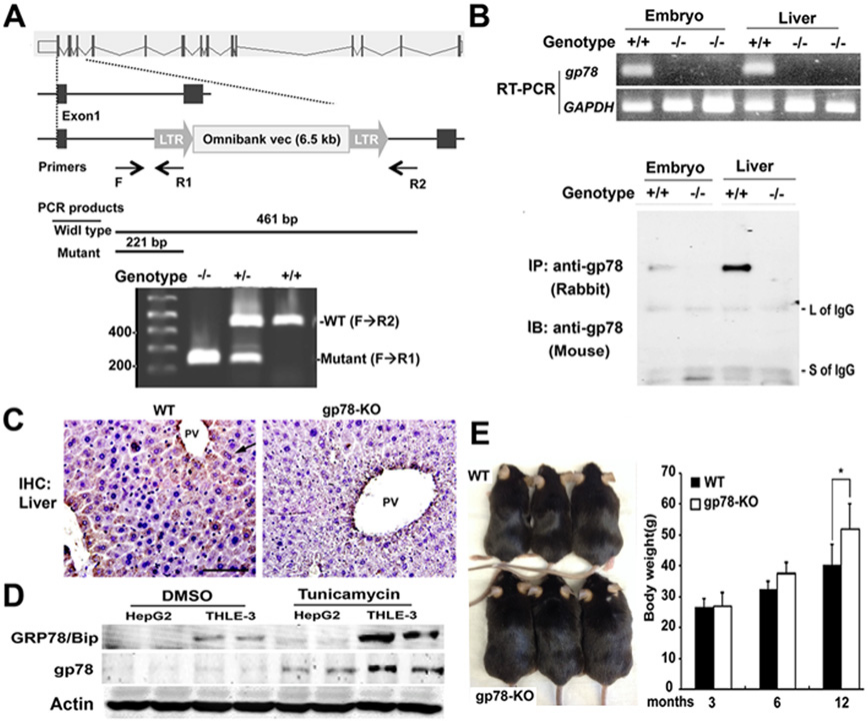
Aged gp78 knockout mice develop obesity and gp78 is unregulated upon ER stress. (**A**) Schematic of gp78-targeting strategy. The gp78 allele disrupted by the insertion of a gene trap vector (OmniBank Vector 76) in the first intron. Genomic DNA isolated from WT, heterozygous and homozygous was genotyped by PCR. Primer set including double reverse primers was designed for internal PCR quality. (**B**) Total RNA and lysates were prepared in embryo and adult liver and were analyzed in RT-PCR (Top), both lysates were immunoprecipitated with rabbit anti-gp78 antibodies (epitope: 524–537) and then immunoblotted with monoclonal anti-gp78 antibodies (epitope: 451–551) to identify gp78 protein (bottom). (**C**) Immunohistochemistry (IHC) of liver with monoclonal anti-gp78 antibody. Arrow indicates gp78 positive stain. PV, portal vein. Original image, x400. (**D**) Upregulation of gp78 in response to ER stress. Immortalized THLE-3 and cancerous HepG2 liver cells were treated with tunicamycin (1ug/ml) for 24 hrs. and lysates were immunoprecipitated with anti-gp78 antibody and immunoblotted. GRP78/BIP, a chaperon of UPR pathways. (**F**) Photography of abdomen of 1-year-old mice (left). Comparison of body weight of WT and gp78-KO mice at 3 months (n = 25), 6 months (n = 25), 12 months (n = 25) old (right). Asterisk indicates a significance determined by Student’s test (*, p < 0.05).

Gp78 heterozygous mice were obtained with ES cell line, generated by genomic insertion of a gene-trapping vector. The gene-trapping vector was inserted in the first intron downstream of the ATG codon between the first and second exons, which block production of endogenous transcription (Fig. 1A). To examine the necessity of gp78 during embryonic development, intercrosses between *gp78* ^*+/-*^ heterozygous progenies were performed and yielded viable *gp78* ^***-/-***^ homozygous mice at a normal Mendelian frequency. To rule out the presence of truncated gp78 protein downstream of the disruption site in gp78-KO cells, RT-PCR and protein analyses were performed and confirmed that gp78-KO mice did not express gp78 mRNA or protein in embryonic lysate or liver tissue (Fig. 1B). Hereafter these mice not expressing gp78 are referred to as gp78-KO. Of note, endogenous gp78 protein in wild-type (WT) cells and tissues was not detected in regular Western blots, regardless of maximum loading and usage of various anti-gp78 antibodies. Gp78 protein could be visualized in wild-type mice after enrichment by immunoprecipitation with anti-gp78 antibody, suggesting that gp78 expression is not ubiquitous in hepatocytes and/or is normally very low. Consistently, immunohistochemistry showed that gp78 was stained in wild-type hepatocytes around the portal vein (Fig. 1C). Next, we addressed the regulation of gp78 expression in immortalized liver cells and cancer cells. To examine whether gp78 expression is involved in ER stress-induced ERAD in liver cells, we treated cells with tunicamycin (TM) to induce ER stress, resulting in up-regulated gp78 expression (Fig. 1D), suggesting that gp78 expression and its activity are required to alleviate disruption of ER homeostasis. Crossbreeding with inter- and intra-genotypes showed that homozygotes displayed no obvious phenotypes, as they are viable, grossly normal and fertile. It is surprising that gp78 is not essential for embryonic development or survival, considering the function and importance of ERAD machinery and its constitutive process [16,17].

### Ablation of gp78 induces hepatic steatosis and inflammation in aged mice

**Fig 2:**
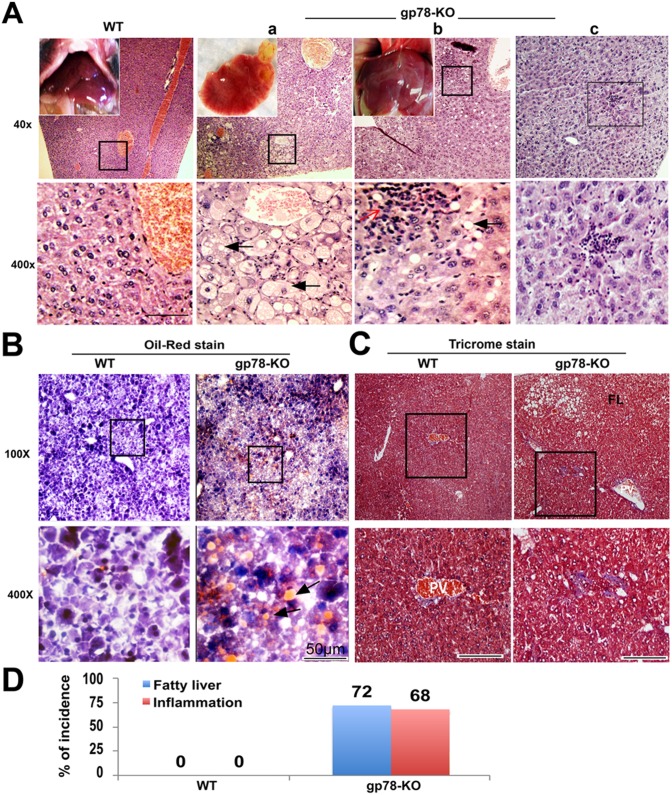
Gp78-KO mice spontaneously develop hepatic steatosis with inflammatory infiltrates. (**A**) Livers of 1-year-old gp78 KO mice, grown with normal diet were stained with H&E and visualized as indicated magnification. (a) Steatohepatitis shows swelling cytoplasm with lipid droplets (black arrow) and infiltrating cells. (b) Mild lipid droplets and infiltrating cells (Red arrow). (c) Infiltrate cells gather in the absence of lipid droplets. (**B**) Oil red stain. Arrow indicates lipid droplets. (**C**) Trichrome stain was preformed to identify blue colored collagen fibers. PV; portal vein, FL; fatty liver area. (**D**) Percentage of incidence including mild fatty liver, inflammation in 1-year-old KO mice (n = 25 mice per each group).

Aging is arguably the most universal risk factor for most of the common diseases, including type 2 diabetes mellitus (T2DM), neurodegeneration and cancer which is frequently associated with metabolic dysregulation and/or accumulation of misfolded protein aggregates [22]. Thus, we expected that aging-related metabolic disorder and dysregulation of protein homeostasis potentiate gp78 loss-associating ERAD deficiency because of proposed gp78 roles *in vitro* as a metabolic regulator and/or a scavenger for misfolded proteins. It was a remarkable observation that more than 70% of gp78-KO mice grow fat with abdominal obesity at the age of around 1 year (Fig. 1E). Visceral adiposity is associated with lipid dysregulation, insulin resistance, and NAFLD [2]. In addition, the liver is the organ responsible for *de novo* lipogenesis and systemically controls acquisition and removal of triacylglycerol (TG), derived from three sources (diet, *de novo* synthesis and adipose tissue). Thus, we explored development of hepatic disease in gp78-KO mice, and examined gene expressions relative to lipid metabolism and ER stress markers in liver tissues of 12-month-old obese gp78-KO mice, resulting in no significant difference of the gene expression pattern (S1A Fig.). According to previous studies in cultured cells with exogenous cholesterol, gp78 ubiquitinates HMGCR, which is a rate-limiting step in cholesterol biosynthesis and also Insig-2, which inhibits activation of SREBPs (SREBP-1a, -1c), promoting transcription of genes relative to lipid biosynthesis and mobilization [23–25]. However, up regulation of HMG-CoA reductase and suppression of SREBP-1 were not significantly observed although Insig-2 up regulation is consistently observed in our gp78-KO hepatocytes. In agreement with a report of other gp78-KO embryonic fibroblast cells [26], gp78-KO mice also show no evidence that gp78 is directly implicated in cholesterol synthesis through HMGCR degradation. Next, we stained with hematoxylin and eosin (H&E) to examine dyslipidemia in several liver lobes of fatty gp78-KO mice. Intriguingly, we observed lipid droplets of hepatocytes along with inflammatory infiltration solely at localized regions of liver lobes and not entire regions (Fig. 2A). Evidence recapitulating NASH in gp78-KO mice indicates that hepatocytes of gp78-KO are significantly enlarged and distended by large single or multiple well-defined droplets of fat, sharply different from the WT hepatocytes that have distinctly round nuclei with one or two prominent nucleoli (Fig. 2A, a). Next, the accumulation of lipids in the liver of gp78-KO mice was confirmed by Oil-red O staining (Fig. 2B). Masson’s trichrome stain for validating progressive NASH revealed an irregular distribution of collagen fibers in the liver of gp78-KO, indicative of liver injury and fibrosis (Fig. 2C). Another noticeable phenotype of gp78-KO mice is hepatic inflammation, characterized by the infiltration of inflammatory cells in which lymphocytes infiltrate, gather, and interface among liver lobules in gp78-KO liver regardless of fat accumulation (Fig. 2A, c and S2 Fig.). Thus this hepatitis of gp78-KO mice is likely to be independent of steatohepatitis progression. In summary, the liver of all-obese gp78-KO mice developed simple steatosis and/or hepatic inflammation although to varying degrees (Fig. 2D). These results demonstrate that gp78 plays a suppressing role in abnormal fat accumulation and its depletion leads to nonalcoholic steatohepatitis (NASH) in aged mice. Meanwhile, various degrees of steatosis according to gp78-KO livers prevented us from identifying molecular relevance to lipogenesis and ER stress in immunoblots (S1A Fig.). However it allows for the possibility that hepatocytes with the same gp78 null genetic background are differentially at risk of steatosis and gp78 deletion by itself is insufficient for NASH progress, which is simultaneously induced by age-dependent ‘second hit’.

### Loss of gp78 under acute ER stress leads to ER stress-mediated steatosis through SREBP-1 activation

**Fig 3:**
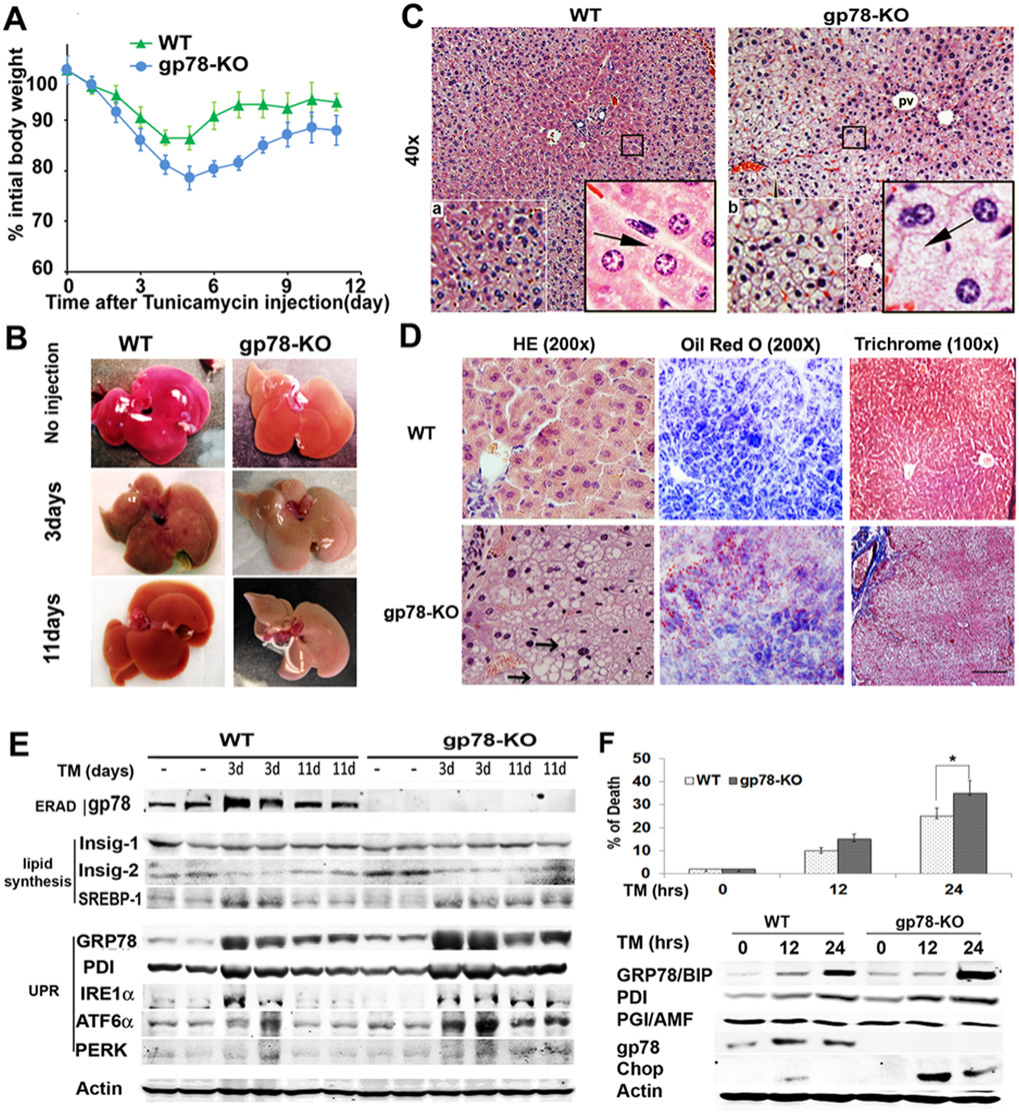
Gp78-KO mice with acute ER stress progresses to severe fatty liver through UPR-driving SREBP-1 activation. (**A**) 1mg/kg body weight of tunicamycin (TM) was intraperitoneally injected to 6 months-old gp78-KO mice. Body weight is represented after normalization to the starting weight as the meant ± SE (n = 3 per group). (**B**) Radish livers are brown colored after TM injection. Liver of gp78-KO at 11 days was not recovered. (**C**) Acute ER stress potentiates entire fatty liver of gp78-KO. H&E stain at 3 days after TM injection. Ballooned cells (b) are typically bigger size than WT hepatocytes (a). Cytoplasmic lipid droplets (arrow) (40x). (**D**) Prolonged fatty liver and fibrosis in gp78-KO. H&E, oil red and Trichrome stains at 11 days after TM injection. White ballooned cells (arrows) were maintained on H&E (200x). Frozen tissues were stained with oil-red O. Irregular fibrosis was extended from connective tissue of portal vein surrounding accumulated lipid droplets (white spots) at pinkish gp78-KO liver (100x). (**E**) Persistent SREBP-1 activation along with UPR up regulation is responsible for fatty liver of gp78-KO. TM-injected mice were scarified respectively (n = 3). Liver extracts at indicated days were subjected to immunoblots. GRP78, Glucose-Regulated Protein; PDI, Protein Disulfide Isomerase, SERBP; Sterol Regulatory Element Binding Transcription Factor; Insig, Insulin Induced Gene. (**F**) Chop-mediated apoptosis. Cell survival was analyzed with viable counting in gp78-KO mouse embryonic fibroblast (MEF) cells treated with TM (1μg/ml) as indicated times (top). Induction of UPR was analyzed in immunoblots and gp78 expression was visualized after its immunoprecipitation (bottom). Chop; ER stress-mediated apoptosis marker.

**Fig 4:**
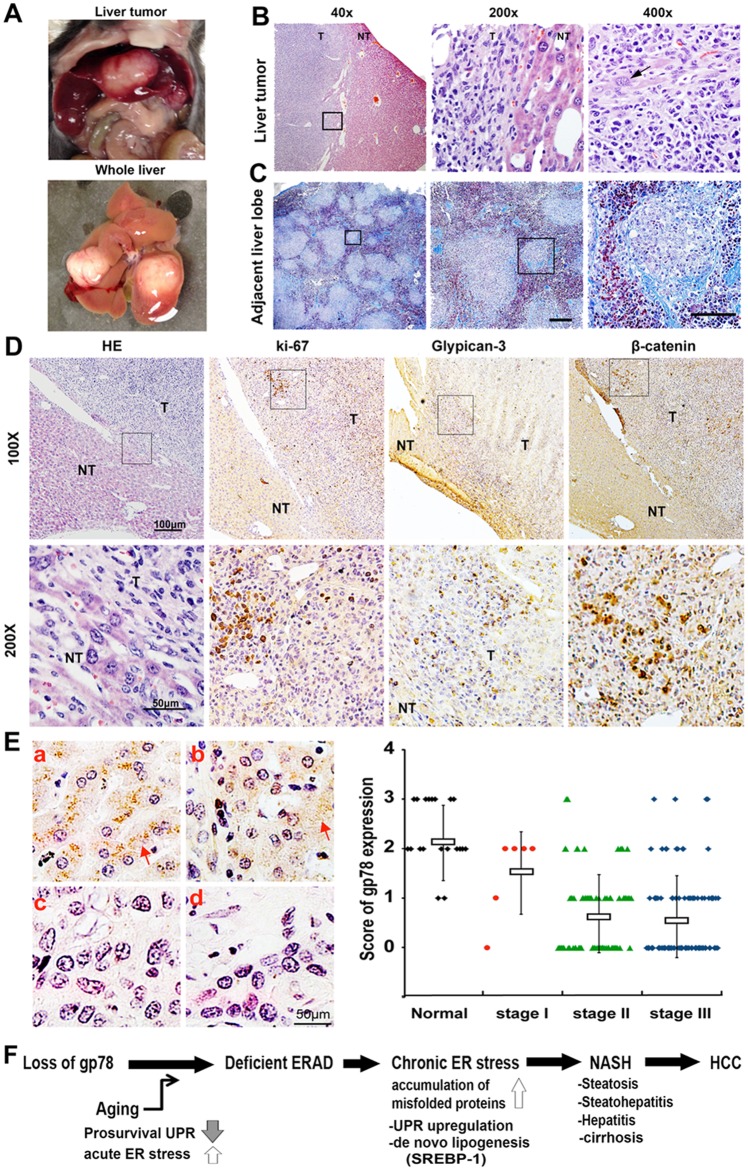
Gp78-KO mice spontaneously develop cirrhosis and liver cancer. (**A**) Photography in gp78-KO mouse harboring two liver tumors. (**B**) H&E stain. Arrow indicates characteristic of hepatic cells and multiple nucleoli per nucleus. (**C**) Cirrhosis in gp78 liver. Trichrome stain in another adjacent lobe of liver bearing tumor. Blue color indicates fibrosis. (**D**) Serial sections of gp78-KO liver harboring tumor (T) and adjacent normal liver tissue (NT) were subjected to IHC analysis with antibodies specific to ki-67, proliferation marker; glypican-3, human HCC marker; β-catenin, aberrant Wnt signaling marker respectively. (**E**) Gp78 down regulation in IHC array of human HCC. Duplicated specimens per a patient were stained with anti-gp78 antibody. A, normal liver (> 50% stain); b, stage I (< 50% stain); c, stage II (<10% stain); d, stage III (0% stain). Graph represents the level of gp78 expression according to tumor grade (normal liver, n of patient = 10; stage I, n = 3; stage II, n = 40; stage III, n = 46). A score was calculated (0, negative; 1, < 10%; 2, 10% to 50%; 3, > 50% positive stained). (**F**) Hypothetic mechanism underlying NASH and HCC pathogenesis in gp78-KO mice. Gp78-mediated ERAD efficiency gives rise to hypersensitivity response to acute ER stress following aging, leading to chronic ER stress in which misfolded proteins are persistent and induce Chop-mediated apoptosis, SREBP-mediated lipogenesis, ultimately leading to inflammation, NASH, and cancer.

We first wanted to understand the mechanism(s) by which knockout of gp78 gives rise to high incidence of NASH in aged mice. As we hypothesized that both gp78 deletion and ‘age-related second hit’ are responsible for NASH development in gp78-KO mice, it drove us to examine whether an age-related second hit is spontaneous and random ER stress, displayed in various grades throughout liver. Thus, we expected that entire gp78-KO liver would accumulate lipid droplets in response to tunicamycin, an acute ER stress inducer. We injected a sub-lethal dosage of tunicamycin (TM) intraperitoneally to 6-month-old gp78-KO mice, which were indistinguishable from WT mice. As expected, the mice rapidly lost weight after TM treatment, with a greater loss in gp78-KO than WT mice (Fig. 3A). TM-injected livers were brown colored at 3 days (Fig. 3B). Liver color of gp78-KO at 11 days did not completely revert to that of controls. Histological analysis visualized a marked difference between the liver of WT and gp78-KO mice after TM injection. While smaller lipid droplets were observed in TM-treated WT liver under high magnification, lipid droplets along with cell ballooning were easily detected throughout the entire gp78-KO liver (Fig. 4C). TM-induced steatosis of gp78-KO displayed in the entire liver, compared to local and partial areas in spontaneous steatosis of aged gp78 KO liver (Fig. 2A and C). It strongly suggested that spontaneous NASH in aged gp78-KO mice is caused by spontaneous and random ER stress. Next, we performed immunoblot analysis with liver lysates to elucidate molecular mechanisms underlying the fatty liver of gp78. In consistent with the work on cell lines (Fig. 1D), The defensive role of gp78 against acute ER stress was implied, based on the observation that gp78 expression was also up regulated *in vivo* after TM treatment (S1B Fig.). Along with this, the GRP78 chaperon, protecting misfolded proteins showed higher expression in TM-injected gp78-KO than WT mice. Previous reports showed that chronic ER stress is involved in the pathogenesis of steatosis [5,10]. The molecular mechanisms underlying ER stress-mediated steatosis include up regulation of transcripts and activation of SREBP-1, which is a master regulator of fatty acid and triglyceride biosynthesis, whereas Insig-2 is an interacting suppressor of SREBP-1 on the ER membrane [24]. Thus, under chronic ER stress, down regulation of Insig-2 leads to SREBP-1 activation followed by *de novo* lipogenesis and liver steatosis [10,24]. We examined down regulation of Insig-2 followed by up regulation of the cleaved form of SREBP-1 under TM stimulus and observed more activation of SREBP-1 in TM-treated gp78-KO liver (S1B Fig.). Thus SREBP-mediated lipogenesis is in part responsible for TM-induced fatty liver of gp78-KO. The next question was whether the slower recovery of body weight in gp78-KO mice (Fig. 3A) is due to prolonged ER stress in which protein misfolding is persistent or excessive. We analyzed 11-day-old mice injected with TM to examine liver status. Results indicate persistent steatosis as well as progression of fibrosis in gp78-KO liver, while WT was fully recovered to their non-injected status (Fig. 3D). Consistently, immunoblots showed that SREBP-1, regulating lipogenesis was still activated at 11 days of gp78-KO liver whereas its level of WT was recovered to the level in untreated mice (Fig. 3E). In addition, TM-treated gp78-KO at 11 days showed longer half-lives of GRP78, PDI, and UPR sensors (IRE, ATF6, and PERK), indirect markers of accumulation of misfolded proteins, than the WT counterparts. Maintained expressions of UPR implied prolong ER stress in TM-exposed gp78-KO liver. When ER function is severely impaired during prolonged ER stress, the organelle elicits apoptotic signals and C/EBP homologous protein (CHOP) is a crucial factor of the components of the ER stress-mediated apoptosis pathway[12]. To examine whether loss of gp78 affects cell viability due to prolong ER stress, we isolated gp78-KO embryonic fibroblast cells (MEFs). We examined whether the viability of gp78-KO MEFs are sensitive to TM treatment (Fig. 3F). Reduction of survival rate in gp78-KO MEF cells are molecularly supported by a slight up regulation of GRP78, PDI, and significant expression of pro-apoptotic CHOP in gp78-KO MEF cells (Fig. 3F). Conclusively, these results convinced us that the loss of gp78 falls into a state of chronic ER stress following intrinsic or extrinsic factors of ER stress in aged mice. In other words, gp78-E3 ligase plays a critical role in ER stress-mediated ERAD in aged hepatocytes.

### Gp78 plays the role of a tumor suppressor in hepatocellular carcinogenesis

NAFLD, especially the aggressive stage of nonalcoholic steatohepatitis (NASH) is associated with an increased risk of liver cancer and retrospective data suggest that about 27% of cases of NASH transform to HCC after the development of cirrhosis, a late stage of scarring (fibrosis) [27]. Interestingly, we observed that overall, >20% (6/25 mice) of the gp78-KO mice developed liver tumors, whereas all WT mice examined were cancer free in the time line examined (Fig. 4A). H&E analysis revealed that these aged mice had dysplastic foci (Fig. 4B and S3 Fig), but we did not observe tumors in other organs, suggesting primary tumor in liver. Next, we examined fibrosis in livers harboring tumors, as cirrhosis is a prerequisite for hepatocellular carcinoma and found severe fibrosis (2/6 mice) in another liver lobe distant from the tumor region (Fig. 4C). To examine the origin of the cancerous tumors, we performed immunohistochemical (IHC) analysis on serial sections using an antibody against ki-67, a proliferating marker and glypican-3, a diagnostic marker for human HCC (Fig. 4D). The etiological link between Wnt signaling and the liver was initially established, through the demonstration that β-catenin activating mutations occur in 20% to 40% of human hepatocellular carcinoma (HCC) [28]. Taking a clue from previous findings about human HCC, we examined whether activation of β-catenin was involved in gp78-KO cancer, resulting in overlapping stained regions with ki-67 in heterogeneous tumor tissue (Fig. 4D). Since gp78 seems to be a tumor suppressor in early tumorigenesis of aged mice, it can be considered as a progression marker and/or potential target in human HCC. Thus, using a tissue array, which contained normal tissues and hepatocellular carcinomas we examined the expression of gp78 in human HCC according to the clinical grade of tumors. We found that gp78 expression of HCC was significantly lower than that of normal liver tissues (Fig. 4E). The analysis in human HCC specimens suggests etiological linking gp78 loss to HCC and putative tumor suppression although the exact molecular mechanisms underlying progression of NASH to cancer are yet to be determined in gp78-KO mice.

## Discussion

In this study, we assessed the key putative suppressor gene responsible for NASH and hepatocellular carcinoma (HCC) as summarized in Fig. 4F. For the first time, the gp78-KO mouse model provides a link of ERAD deficiency to spontaneous NASH and HCC progression, since the phenotype of gp78-KO mice mimic steatosis, steatohepatitis, cirrhosis, and liver cancer of human liver pathogenesis. NASH is a metabolic disease caused by liver lipogenesis and delivery of low-density lipoprotein (LDL) as well as the flow of free fatty acids (FFAs) coming from gut and adipocyte tissue [2]. It remains to be elucidated how gp78 loss in intestine and gp78-KO obesity affects NASH, although we showed that chronic ER stress and SREBP-mediated *de novo* lipogenesis in liver is responsible for NASH of gp78-KO (Fig. 3E and S1B Fig.). Noticeably, this observation of gp78 null mice contradicts the previous report showing that liver-specific gp78-KO mice are resistant to normal chow diet and Western-type diet-induced obesity due to suppressed SREBP by up regulation of its negative regulators, Insig-1/-2 [29]. Although it is not easy to reconcile molecular mechanisms in both mice, possible explanations include that gp78 knockout in whole mice might overcome metabolic stress observed in liver-specific gp78-KO during embryonic developmental stages and early growth. Thus, the opposite phenotype in both mice is required to be further confirmed through suppression and add-back studies, including cross-breeding and *in vitro* cell culture. Meanwhile, as gp78-KO derived NASH occurs in an age-dependent fashion, it might be potentiated due to age-linked declines in expression and activity of key ER molecular chaperones and folding enzymes which compromise proper protein folding and the adaptive response of the UPR [30]. Simple hepatitis (Fig. 2A, c and S2 Fig) was observed in gp78-KO livers which did not show steatosis. This might provide evidence for the proposed risk of ERAD deficiency in which defective secretory and transmembrane proteins are released or exposed at the cell surface to interfere with cell-cell communication and promote autoimmunity by the recognition of self-antigen [31].

As gp78 contributes to tumor progression as reported in sarcoma metastasis by targeting KAI1 for degradation [32] and mammary gland hyperplasia by MMTV-driven 78 transgenic overexpression [33], gp78 is likely to play an opposing roles of tumor suppression or promotion according to tissue context and tumor stages. Although gp78 plays crucial roles in liver homeostasis and suppresses the development of liver tumor, tumor-promoting or suppressing gp78 roles remain clear in human liver carcinomas etiologically arising from various causes. *In vitro*, cell-based studies showed that AMF is a target ubiquitinated by gp78 [34] and AMF/PGI protection against ER stress is mediated by gp78/AMFR receptor [35]. However, we did not observe supporting evidence of AMF/PGI up regulation in liver and MEF cells of gp78-KO in previous findings (Data not shown).

ERAD is involved in UPR for ER homeostasis by eliminating misfolded proteins [36], but the knockout model of ERAD has not been established with respect to NASH and HCC progression. Since the Hrd1/synoviolin of gp78 homologue and gp78-interacting ATPase p97 knockout mice are embryonically lethal [16,17], link of ERAD to NASH and liver cancer could not be uncovered. But gp78 is not essential for embryonic development based on the birth and survival of gp78-KO mice. Ironically, gp78-KO mice escaping threat and natural selection at the developmental stage are likely to generate age-linked liver disease. Our finding is consistent with protective role of gp78 against ER stress in zebra fish liver [37].

Research progress has elucidated the positive role of UPR to survival in cancer which has the risk of chronic ER stress due to a fast growth rate and leads to poor vascularization of tumor mass, low oxygen supply, nutrient deprivation, and pH changes [36,38]. The related findings have raised the possibility of targeting the UPR components as an effective strategy for cancer therapy and overcoming drug resistance. Meanwhile, it has been proposed that the inhibition of ERAD induces UPR, which efficiently restores cellular homeostasis, shifting its survival function towards proapoptotic aspects of the UPR. On the contrary, our findings that gp78 acts as liver specific tumor suppressor, opens an unexplored research window to study an etiological link of ERAD for disease prevention and better outcomes in human patients; it might be clinically significant that normal regulation of ERAD machinery or its enhancement are accomplished by the maintenance of gp78 expression and activity. Of note, the data document the role of gp78 in NASH. The association to NAFLD will further be determined experimentally.

## Materials & Methods

### Generation of gp78 null mice

Mutant gp78 mice were generated using a gene-trapping technique [39]. Mice (strain C57BL/6) were cloned from an ES cell line (IST12797A11; Texas Institute for Genomic Medicine, TIGM). The ES cell clone contained a retroviral insertion in the gp78 gene identified from the TIGM gene trap database and was microinjected into C57BL/6 blastocysts following standard procedures. The OmniBank Vector 76 contained the selectable marker β-geo, a functional fusion between the β-galactosidase and neomycin resistance genes, for identification of successful genomic insertion. Chimeric males were bred with C57BL/6 females for germ line transmission of the mutant gp78 allele.

### Animal experiments

All protocols for mice were reviewed and permitted by the University Committee on Use and Care of Animals at Wayne State University, including provisions for minimizing animal distress and maximizing animal well-being that conform to NIH guidelines. Mice were fed standard rodent chow and housed in a controlled environment with 12 hrs. light and dark cycles. Littermate controls were used and age and gender were matched for all experiments. Mice were injected intraperitoneally with 1mg/kg body weight of TM or vehicle and then liver were isolated on the designated day.

### Antibodies and Reagents

Gp78 mouse monoclonal antibody was purchased from Abnova. Polyclonal anti-gp78 antibodies were generated in immunized rabbit with recombinant protein of gp78 cytosolic domain (309–643 aa) and peptide (524–537 aa), and purified with affinity chromatography. Anti-insig-1/-2, anti-SREBP-1, anti-HMGCR, and anti-fatty acid synthase, anti-GRP78 antibodies were obtained from Santa Cruz. Anti-PDI and anti-Chop were obtained from Cell Signaling Technology. Anti-actin and Oil-red O was purchased from Sigma. Masson’s Trichrome Stain Kit was purchased from Polysciences.

### RNA, Western blotting and immunoprecipitation

Western blotting and immunoprecipitation was previously described [40,41]. Tissue was homogenized with an electronic homogenizer in RIPA buffer including protease inhibitors (Roche) for liver lysates or in Trizol RNA reagent (Invitrogen) for total RNA. Immunoprecipitation with antibody and lysates was carried out overnight. Gp78 protein was visualized in immunoblots after its immunoprecipitation and enrichment. Each experiment was repeated at least twice.

### Histological and Immunohistochemical Analysis

Liver specimens were fixed in 10% formalin; the sections were stained with hematoxylin and eosin (H&E). For IHC, five micrometer sections were obtained from formalin fixed, paraffin-embedded tissue blocks and then deparaffinized, rehydrated, and microwaved in 1 mmol/L sodium citrate buffer, pH 6.0. The sections were washed 3× in PBS and blocked with Super Block (Skytek Laboratories) for 10 minutes. Sequential sections were then incubated in PBS and linked with biotinylated primary antibodies at 4°C overnight. The sections were then washed 3× for 10 minutes each in PBS and linked with biotinylated secondary antibodies followed by DAB development (Vector Laboratories). Images were documented with an OLYMPUS BX40 microscope (Olympus). Liver carcinoma and normal tissue array (BC03118) consisting of normal liver tissue and liver carcinoma tissues, duplicate cores per case were purchased from US Biomax (Rockville, MD). Four micrometers of serial sections from the tissue array were processed for immunohistochemical analysis using anti-gp78 antibodies (Abnova) antibodies.

### Isolation of MEFs

Mouse embryonic fibroblasts (MEFs) were isolated from E12.5 embryos of the gp78^-/-^ and wild type mice by enzyme digestion after elimination of the head and internal organs. The isolated cells were cultured in Dulbecco’s modified Eagle’s medium containing 20% fetal calf serum.

### Statistical analysis

Data are presented as means ± SEM. Differences between groups were evaluated for statistical significance using Student’s t-test. P values less than 0.05 were regarded as significant.

## Supporting Information

S1 Fig Loss of Gp78 induces highly UPR pathways under acute ER stress. (**A**) There is no significant difference of ER stress and lipogenesis markers in whole liver lysates of WT and obese gp78-KO mice under normal diet. Liver tissues of gp78 mice were analyzed in immunoblots as indicated ages (N = 2 or 3). Lipogenesis includes FAS, fatty acid synthase; HMGCR, HMG-CoA reductase, regulating cholesterol synthesis; SREBP-1regulating *de novo* lipogenesis, Sterol Regulatory Element Binding Transcription Factor 1; Insigs suppressing SREBP, Insulin Induced Gene 1. ER stress markers, GRP78 chaperon, Glucose-Regulated Protein; PDI foldase, Protein Disulfide Isomerase. (**B**) gp78-KO livers highly express UPR pathways and matured (m)-SREBP-1, which induces lipogenesis along with inverse correlation of Insig-2 under acute ER stress (tunicamycin, TM). Mice were injected with TM and scarified for liver lysates at 1 day after injection (n = 3 per each group).
(TIF)

S2 Fig Aged gp78-KO mice spontaneously develop hepatitis. HE stains representing hepatitis of gp78-KO show infiltrated immune cells around hepatocytes (case 1), portal vein (case 2), or damaged liver (case 3 and 4) in gp78-KO mice. Each case is from a different mouse. Arrow indicates mild lipid droplets. Focal intralobular necroses along with inflammatory infiltrates in the middle zone of liver lobule (Case 3). Scale bar, 50 μm.
(TIF)

S3 Fig Hepatocellular tumors were frequently developed in around 1-year-old gp78-KO mice. H&E stains of liver tumors from around 1-years old gp78-KO mice. Case1; tumor at severe fatty and damaged liver, case 2; multiple tumors in mild fatty liver, case 3; sing tumor in non-fatty liver, case 4; liver tumor and adjacent tissue. Case 1, 3 displayed intra-tumor histology and case 2, 4 represent the margin between tumor and adjacent normal tissue. Representative images are shown. Scale bar, 50 μm.
(TIF)
