# Supplementary figures and images for: Gp78, an E3 Ubiquitin Ligase Acts as a Gatekeeper Suppressing Nonalcoholic Steatohepatitis (NASH) and Liver Cancer
Source: PLoS One. 2015 Mar 19;10(3):e0118448. doi: 10.1371/journal.pone.0118448 (PMC4366401; doi:10.1371/journal.pone.0118448)

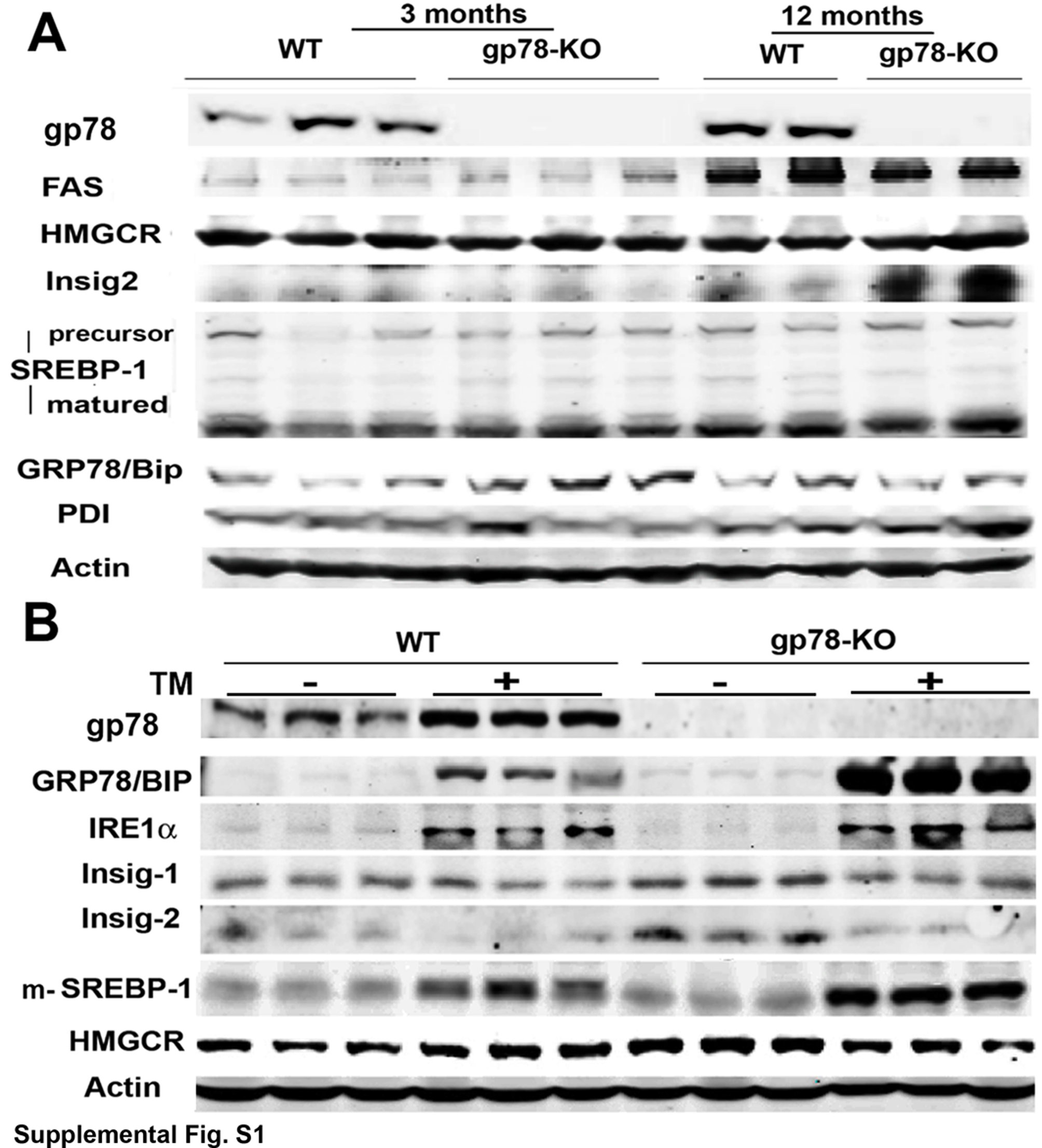

Supplement: S1 Fig — (A) There is no significant difference of ER stress and lipogenesis markers in whole liver lysates of WT and obese gp78-KO mice under normal diet. Liver tissues of gp78 mice were analyzed in immunoblots as indicated ages (N = 2 or 3). Lipogenesis includes FAS, fatty acid synthase; HMGCR, HMG-CoA reductase, regulating cholesterol synthesis; SREBP-1regulating de novo lipogenesis, Sterol Regulatory Element Binding Transcription Factor 1; Insigs suppressing SREBP, Insulin Induced Gene 1. ER stress markers, GRP78 chaperon, Glucose-Regulated Protein; PDI foldase, Protein Disulfide Isomerase. (B) gp78-KO livers highly express UPR pathways and matured (m)-SREBP-1, which induces lipogenesis along with inverse correlation of Insig-2 under acute ER stress (tunicamycin, TM). Mice were injected with TM and scarified for liver lysates at 1 day after injection (n = 3 per each group). (TIF) [file pone.0118448.s001.tif]

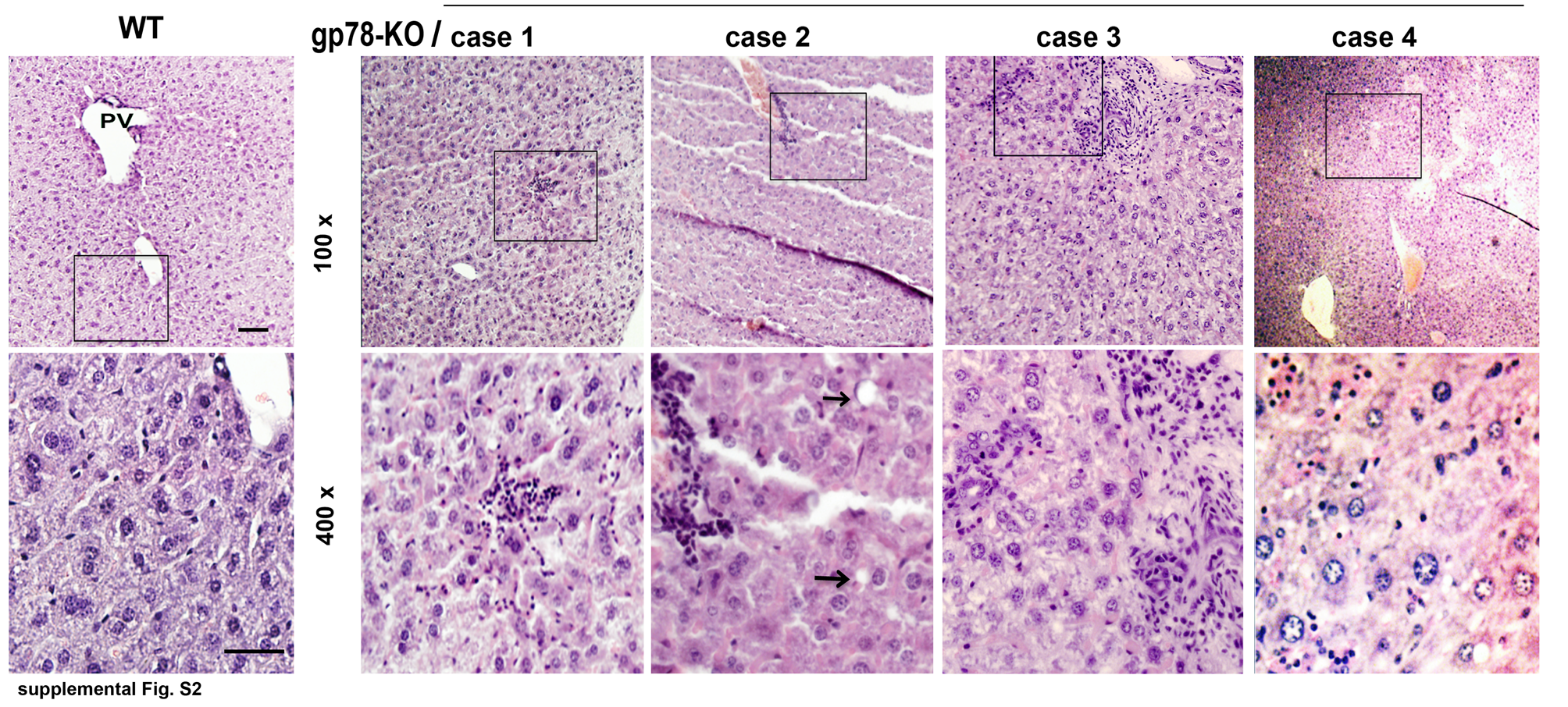

Supplement: S2 Fig — HE stains representing hepatitis of gp78-KO show infiltrated immune cells around hepatocytes (case 1), portal vein (case 2), or damaged liver (case 3 and 4) in gp78-KO mice. Each case is from a different mouse. Arrow indicates mild lipid droplets. Focal intralobular necroses along with inflammatory infiltrates in the middle zone of liver lobule (Case 3). Scale bar, 50 μm. (TIF) [file pone.0118448.s002.tif]

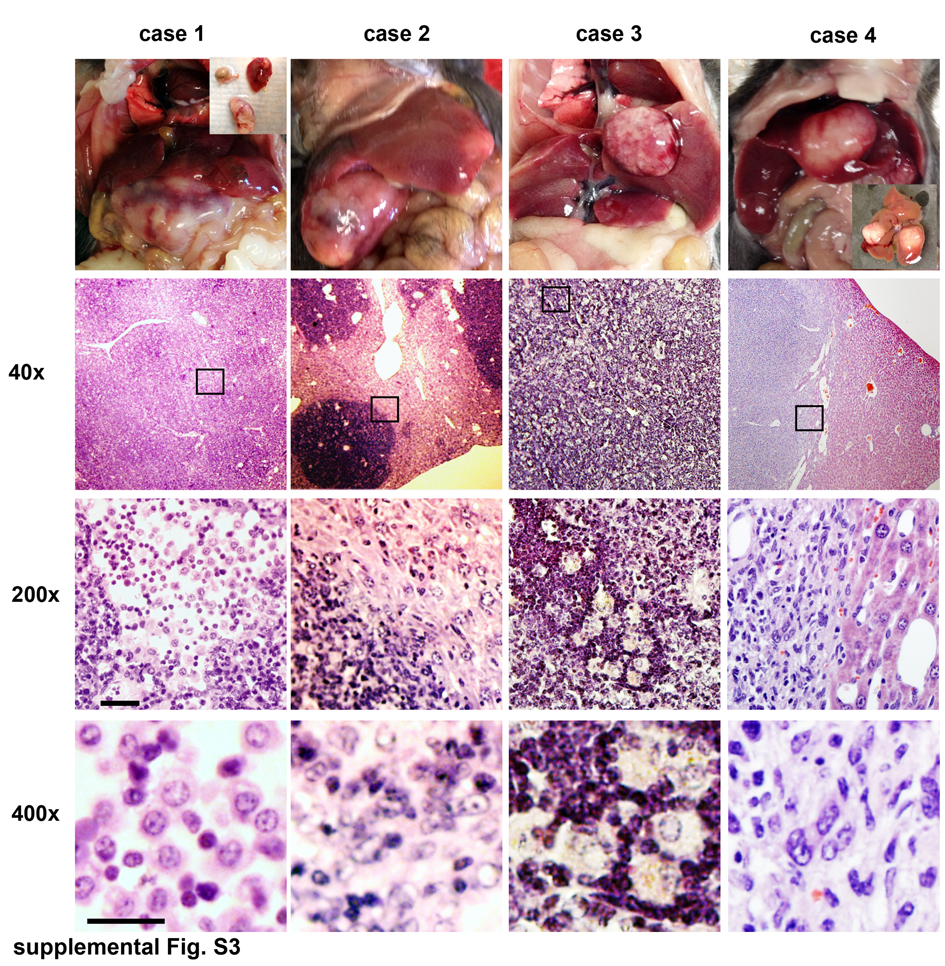

Supplement: S3 Fig — H&E stains of liver tumors from around 1-years old gp78-KO mice. Case1; tumor at severe fatty and damaged liver, case 2; multiple tumors in mild fatty liver, case 3; sing tumor in non-fatty liver, case 4; liver tumor and adjacent tissue. Case 1, 3 displayed intra-tumor histology and case 2, 4 represent the margin between tumor and adjacent normal tissue. Representative images are shown. Scale bar, 50 μm. (TIF) [file pone.0118448.s003.tif]
